# Exposure to disaster information on social media, depressive symptoms, and alcohol and cannabis use in the aftermath of two natural disasters

**DOI:** 10.1016/j.abrep.2026.100723

**Published:** 2026-06-24

**Authors:** Francis Dalisay, Jay Hmielowski, Young-Rock Hong

**Affiliations:** a College of Journalism and Communications, University of Florida, Gainesville, FL 32611, United States of America; b School of Medicine, Department of Family and Preventive Medicine, Emory University, Atlanta, GA 30322, United States of America

**Keywords:** Natural disasters, Social media, Depression, Alcohol, cannabis

## Abstract

**Background:**

Individuals who directly experience major natural disasters are at an increased risk for developing psychological distress and engaging in substance use. Beyond such direct experiences, however, viewing media portrayals of disasters could serve as an indirect means of exposure to a disaster. The objective of this study was to examine the associations between exposure to natural disaster information on social media, depressive symptoms, and the use of alcohol and cannabis.

**Methods:**

We analyzed cross-sectional data from two separate surveys. The first survey was conducted during the aftermath of Hurricanes Helene/Milton in 2024 in Florida, Georgia, North Carolina, and South Carolina. The second survey was conducted in Los Angeles County after the area's wildfires in 2025.

**Results:**

Findings showed that for both the hurricanes and wildfires surveys, exposure to disaster-related information on social media and depressive symptoms were both positively associated with the use of alcohol and cannabis. Exposure to disaster-related information on social media was positively associated with depressive symptoms, but this finding occurred only in the wildfires survey and not in the hurricanes survey. Finally, there was an indirect association between exposure to disaster-related information on social media and the use of alcohol and cannabis through depressive symptoms. However, this finding too occurred only in the wildfires survey.

**Conclusions:**

For those who directly experience a disaster, there is the potential that using social media for news or information about the disaster may serve as a risk factor associated with heightened distress and substance use.

## Highlights

• Viewing portrayals of disasters in the media may serve as an indirect means of exposure to a disaster.
• Exposure to disaster information on social media positively relates with depressive symptoms.
• Exposure to disaster information on social media positively relates with alcohol and cannabis use.
• Depressive symptoms mediates the link between exposure to disasters on social media and alcohol/cannabis use.

## 1 Introduction

Natural disasters—particularly hurricanes and wildfires—have had devastating impacts on communities around the United States. Research has shown that individuals who have firsthand, direct experiences with natural disasters are at an increased risk for developing forms of psychological distress, including depression (Garfin, Thompson, Holman, Wong-Parodi, & Silver, 2022; Nwankwo et al., 2021; Zhang et al., 2022). Experiencing a major disaster has also been associated with a heightened risk of substance use (Cerdá, Tracy, & Galea, 2011; İspir, Danışman, & Katar, 2024).

Direct experiences that result from disasters could include stressful events such as having one's home damaged or destroyed, losing sentimental possessions, or developing health problems (Garfin et al., 2022; To, Eboreime, & Agyapong, 2021). Yet beyond such direct experiences, viewing media portrayals of disasters could serve as an indirect means of exposure to a disaster (Houston, Spialek, & First, 2018). Depictions of disasters in the media frequently contain distressing images of homes or properties that have been damaged or destroyed (Shin, Fahmy, & Lewis, 2012) and narratives of victims who have experienced serious losses and injuries (Houston et al., 2018; Widmar, Rash, Bir, Bir, & Jung, 2022). In today's media landscape, social media particularly facilitate immediate, continuous, and often unfiltered access to disaster imagery and personal accounts, creating a more immersive and potentially distressing exposure experience compared to curated traditional news coverage (e.g., Sovacool, Xu, Zarazua De Rubens, & Chen, 2020; Stimpson, Srivastava, Tamirisa, Kaholokula, & Ortega, 2025).

Yet more research is warranted to understand whether being indirectly exposed to natural disaster information through social media would result in similar outcomes as direct exposure. To this end, the present study seeks to achieve three goals. First, we examine whether exposure to natural disaster-related information on social media is positively associated with depressive symptoms, on the one hand, and the use of alcohol and cannabis, on the other hand. Second, we test whether depressive symptoms would be positively related with the use of alcohol and cannabis. Finally, we examine a mediation model whereby exposure to disaster-related information on social media is proposed to positively associate with the use of alcohol and cannabis through higher levels of depressive symptoms.

We test our hypotheses by analyzing two sets of survey data. The first dataset was collected during the aftermath of Hurricanes Helene and Milton in 2024, among counties impacted in Florida, Georgia, North Carolina, and South Carolina. The second dataset was collected in Los Angeles County following the Palisades and Eaton fires in 2025. These two datasets allow us to test whether our findings can be replicated across two distinct types of disasters in different parts of the U.S.

### 1.1 Media exposure to disasters, psychological distress, and substance use

The extant disaster-related research has suggested that exposure to disaster information through the media could heighten forms of psychological distress (Juarez et al., 2025; Thompson, Holman, & Silver, 2019), including depression (Pfefferbaum, Nitiéma, & Newman, 2021). For example, Garfin et al. (2022) conducted a longitudinal, 3-wave survey study of Florida residents and found that exposure to media coverage of two hurricanes (Irma and Michael) was associated with symptoms of global distress, which included depression and anxiety. Garfin et al.'s study, however, did not parse out potential differences between traditional media and social media. In another study, Goodwin, Palgi, Hamama-Raz, and Ben-Ezra (2013) showed that using social media to learn about a hurricane (i.e., Hurricane Sandy) was associated with increases in reported post-traumatic stress.

Disaster-related social media exposure may represent a particularly potent pathway to heighten distress, especially considering previous research has suggested that user-generated disaster content on social media tends to be emotionally charged and often unfiltered (Sovacool et al., 2020; Stimpson et al., 2025). Moreover, beyond the context of natural disasters, recent meta-analyses have indicated that social media use itself is positively associated with depression (Cunningham, Hudson, & Harkness, 2021; Yoon, Kleinman, Mertz, & Brannick, 2019). Thus, based on the expectation that individuals can indirectly experience natural disasters through social media, which may then result in heightening distress, we propose the following hypothesis:

*H1: Exposure to disaster information on social media will be positively associated with depressive symptoms.*

Previous studies have also shown that directly experiencing major natural disasters could result in the increased use of various substances, such as alcohol and cannabis (Cerdá et al., 2011; Hakim, McDonald, Lochman, Power, & Vernberg, 2022; Rohrbach, Grana, Vernberg, Sussman, & Sun, 2009). The underlying logic is that individuals who directly experience disasters look for ways to cope. Individuals may engage in maladaptive coping behaviors, such as using various substances like alcohol, tobacco, or other drugs (Alexander & Ward, 2018). The idea of seeking out substances to cope with distress or alleviate mental health symptoms (e.g., depressive symptoms) has been referred to as the self-medication hypothesis (Khantzian, 1997).

Yet individuals can vicariously experience distressing events through viewing them in the media (Tynes, Willis, Stewart, & Hamilton, 2019). This conceptual framework supports the possibility that indirect experiences with disasters, facilitated through consuming disaster-related content on social media, could similarly be related with the use of substances. Apart from the specific context of natural disasters, extant research has linked social media use with alcohol consumption (Alhabash, Park, Smith, Hendriks, & Dong, 2022), tobacco product use (Pokhrel et al., 2021), and cannabis initiation (e.g., Kelleghan et al., 2020). Therefore, it is possible that exposure to disaster information on social media could represent a trigger for substance use (alcohol and cannabis use) as a maladaptive coping mechanism. Thus, our study tests the following hypothesis:

*H2: Exposure to disaster information on social media will be positively associated with the use of a) alcohol and b) cannabis.*

Forms of psychological distress are well-documented risk factors linked with increases in substance use after experiencing a disaster (Flory, Hankin, Kloos, Cheely, & Turecki, 2009; Noel et al., 2021). Studies particularly show that depression and/or depressive symptoms are associated with using alcohol (Conner, Pinquart, & Gamble, 2009) and cannabis (Gorfinkel, Stohl, & Hasin, 2020). Given the link between psychological distress and substance use, we also test the following hypothesis:

*H3: Depressive symptoms will be positively associated with the use of a) alcohol and b) cannabis.*

The literature up to this point suggests that there could be an indirect relationship between exposure to disaster information on social media and substance use through depressive symptoms. Alexander and Ward's (2018) conceptual model for post-disaster substance use suggests a sequential process: individuals experiencing stressful events as a result of a natural disaster develop symptoms of depression, which then lead to maladaptive coping strategies, such as substance use. As we explained, individuals can indirectly experience natural disasters through social media. Such indirect experiences with disasters through social media may then result in heightened distress, such as depression. Subsequently, heightened distress would be associated with substance use as a coping strategy. While few studies have specifically examined the indirect association between exposure to disaster information on social media with substance use through depression, scholars have extensively looked at intervening variables between exposure to content in the media and important outcomes such as people's attitudes and behaviors (Hayes, Preacher, & Myers, 2014). Cénat, Blais, Lavoie, Caron, and Hébert (2018), for instance, found evidence that experiencing traumatic events online (i.e., cyberbullying) increased substance use through higher levels of psychological distress. Thus, we propose our final hypothesis:

*H4: Exposure to disaster information on social media will be positively associated with the use of a) alcohol and b) cannabis through depressive symptoms.*

## 2 Methods

### 2.1 Study design and sample

This cross-sectional study, approved by the Institutional Review Board (IRB) at the REDACTED (approval numbers: REDACTED, REDACTED), utilized online surveys targeting residents of counties with FEMA disaster declarations in Georgia, North Carolina, and South Carolina following Hurricane Helene and in Florida following Helene/Milton in 2024, and residents of Los Angeles County, following the wildfires in 2025. We contracted Qualtrics (2025) to collect the data and administer both surveys. The surveys took approximately 15–20 min to complete. Informed consent was obtained through asking participants on the first page of the survey whether they consented to completing it. Participants were excluded from analyses based on three criteria: (1) failure to provide informed consent, (2) not residing in an impacted county (i.e., a hurricane-affected county for the Hurricanes survey, or Los Angeles County for the Wildfires survey), and (3) completing the survey in less than half the median survey completion time (i.e., less than 4.65 min; median = 9.3 min). The survey vendor implemented this speed check exclusion as standard protocol to protect data quality and minimize the influence of careless or inattentive responding. Excessively rapid survey completion is a well-established indicator of satisficing behavior, in which respondents provide cursory or random answers rather than thoughtfully engaging with survey items (Curran, 2016; Meade & Craig, 2012). This concern is especially salient in the present study, given the sensitive nature of the constructs assessed, including substance use and disaster exposure, for which low-quality responses could introduce meaningful measurement error and bias study estimates. The use of one-half the median completion time as a minimum response threshold is consistent with recommended best practices in survey methodology for identifying and excluding insufficient effort responding (Curran, 2016).

Furthermore, to provide transparency regarding participant flow, we have included a CONSORT-style diagram for the Hurricanes survey detailing the number of individuals invited to participate (*n* = 1283), excluded due to incomplete survey completion (*n* = 516), excluded due to not meeting the speed check criterion (*n* = 43), and ultimately included in analyses (*n* = 724). For the Wildfires survey, the online panel vendor (Qualtrics) was unable to disclose the number of individuals invited to participate; therefore, the participant flow diagram for this sample reports the number of individuals included in analyses (*n* = 750) and excluded due to not meeting the speed check criterion (*n* = 246).

### 2.2 Surveys

#### 2.2.1 Hurricanes survey

Surveys were administered in October 2024, or one month after Hurricane Helene made landfall, in Georgia, North Carolina, and South Carolina. In Florida, the survey was administered a month after Hurricane Milton made landfall, or November 2024. Quota sampling was used to ensure representation across demographic characteristics, including age and gender in the hurricanes survey. After excluding participants based on the exclusion criteria described above, the final sample size used for the analyses was *n* = 1318.

#### 2.2.2 Wildfires survey

A survey was administered in Los Angeles County in the last week of March 2025, which was about 8 weeks after the Eaton Fire was fully contained. Quota sampling was also used for the wildfires survey. After exclusion, the final sample size used for the analyses was *n* = 748.

### 2.3 Measures

#### 2.3.1 Independent variable

**Exposure to disaster information on social media**. We asked participants in the hurricanes survey, “In the past month, how often did you use the following social media platforms to obtain information related to Hurricane Helene/Milton and recovery efforts?” For the wildfires survey, we asked participants, “In the past month, how often did you use the following social media platforms to obtain information related to the LA-area wildfires and recovery efforts?” We included the following social media platforms: (a) Facebook, (b) Instagram, (c) X (formerly known as Twitter), (d) YouTube, (e) WhatsApp, (f) TikTok, (g) Reddit, (h) Nextdoor (Nextdoor: Your Neighborhood), and (i) Ring Community. Responses were measured along a 5-point scale (1 = never, 5 = very often), then averaged and combined to form a single measure.

#### 2.3.2 Dependent variables

**Alcohol and cannabis use***.* Participants' alcohol and cannabis use were assessed with the following respective questions: “In the past 30 days (i.e., 1 month), on how many days have you had a drink containing alcohol?” and “In the past 30 days (i.e., 1 month), on how many days did you use marijuana?” For the latter item, we specified that the term “marijuana” referred to cannabis that contains THC. These items were adapted from the Behavioral Risk Factor Surveillance System (2022). Responses were measured along a 7-point scale (1 = 0 days, 2 = 1–2 days, 3 = 3–5 days, 4 = 6–9 days, 5 = 10–19 days, 6 = 20–29 days, 7 = daily).

#### 2.3.3 Mediator

**Depressive symptoms.** We used four items from the Center for Epidemiologic Depression Scale (CES—D) (Carleton et al., 2013) to measure depressive symptoms. The items asked participants how often within the last week (7 days), have they experienced the following: (a) felt sad, (b) felt that you could not shake off the blues, even with help from your family or friends, (c) felt depressed, and (d) had difficulty falling asleep (1 = less than one day, 4 = 5 to 7 days). Responses were averaged and combined to form a single measure.

#### 2.3.4 Covariates

We measured and controlled for age (continuous), biological sex (1 = male), educational attainment (1 = high school diploma or GED, 8 = Doctorate degree), income (1 = $19,999 or less, 11 = $190,000 and above), race (White = 1, Others = 0), and political orientation (1 = very conservative, 5 = very liberal). For the hurricanes survey, we also included state as a covariate (Georgia, North Carolina, South Carolina), where Florida was treated as the reference group and omitted as a state predictor in all the regression models discussed in our analytical strategy below. We thus included only Georgia, North Carolina, and South Carolina as covariates in the regression models for the hurricanes dataset.

We also measured and controlled for *disaster-related stressors.* We asked participants whether in the past month, they had experienced the following 10 disaster-related stressors as a result of Hurricanes Helene/Milton or the LA wildfires: (a) lack of any resource [food, water, shelter, electricity] for over a week, (b) loss of sentimental possessions or pets, (c) self or household member had health problems, (d) financial loss, (e) lost access to any needed medical care since the hurricane/wildfire, (f) lost access to any regular medications due to the hurricane/wildfire, (g) experienced any delays or difficulties in getting medical appointments since the hurricane/wildfire, (h) forced to change healthcare providers due to facility closures or damage, (i) housing situation changed as a result of the hurricane/wildfire, and (j) experienced any structural damage to their homes due to the hurricane/wildfires. We adapted the first 4 items listed above from Lowe et al. (2015) and we created the 2 remaining items. Responses to all the items for disaster-related stressors were scored with 1 = yes, 0 = no, then averaged and combined to form a single measure.

Finally, since we were specifically interested in the role of social media, we measured and controlled for *exposure to disaster information on other media.* We asked participants how often they use the following sources within the past month to obtain information regarding the hurricanes/wildfires and the post-hurricanes/wildfires recovery: (a) local newspapers, (b) national newspapers (e.g., New York Times, Washington Post, USA Today), (c) local radio news, (d) local TV news, (e) The Weather Channel, and (f) podcasts (national or local) (1 = never, 5 = very often). Responses were averaged and combined to form a single measure.

### 2.4 Analytical strategy

SPSS version 30 was used to analyze descriptive statistics. To test the hypotheses, we utilized OLS regression in Mplus (8.11). Across both datasets, we examined the relationships and indirect relationships for social media use through our mediator on use of both substances in one model (one model per dataset). When estimating our indirect relationships, we utilized 5000 bootstrap estimates to generate our results. We report unstandardized coefficients along with standard errors when reporting the relationships between variables. For our indirect relationships, we report unstandardized coefficients along with 95% confidence intervals. Listwise deletion was used to deal with missing data. All statistical tests were two-sided with a significance level of 0.05.

## 3 Results

### 3.1 Descriptive statistics

Descriptive statistics for demographics and key variables, including their reliabilities, are reported in Table 1. Of note, for both the hurricanes and wildfires datasets, the proportion of females was larger than that of males (hurricanes survey: Male 45%, Female 55%; wildfires survey: Male 48%, Female 52%), suggesting an under-sampling of males. In addition, for the wildfires survey, there appeared to be an under-sampling of Hispanic participants, as 23% of the survey's sample identified as Hispanic, while the U.S. Census (2020) data estimated that about 49% of LA County residents identify as Hispanic/Latino.

**Table 1:** Descriptive statistics for demographics and key variables among participants.

	**Hurricanes survey (n = 1318)**	**Wildfires survey (*n* = 748)**
Gender	Male 45%, Female 55%	Male 48%, Female 52%
Age	46.13 (17.32)	46.10 (17.38)
State	Florida (54%)	–
	Georgia (12%)	–
	North Carolina (20%)	–
	South Carolina (14%)	–
Education	Median “one or more years of college”	Median “Associate's degree (for example AA, AS)”
Income	Median $40,000–$59,999	Median $60,000–$79,999
Ethnicity	White (63%)	White (44%)
	Black (21%)	Black (16%)
	Hispanic (5%)	Hispanic (23%)
	Asian (1%)	Asian (13%)
	Other (10%)	Other (5%)
Political orientation	Very conservative (17%)	Very conservative (12%)
	Conservative (21%)	Conservative (16%)
	Moderate (39%)	Moderate (39%)
	Liberal (14%)	Liberal (20%)
	Very liberal (9%)	Very liberal (13%)
	2.77 (1.15)	3.06 (1.17)


	M(SD)	Cronbach's alpha	M(SD)	Cronbach's alpha
Exposure to disaster information on social media	2.19 (0.91)	0.77	2.40 (1.15)	0.92
Depressive symptoms	2.17 (0.95)	0.85	2.06 (0.94)	0.91

Table 2 reports descriptive statistics for use of both alcohol and cannabis among participants for both the hurricanes and wildfires datasets. On balance, the trends in the distribution of alcohol and cannabis use among both datasets were similar, with less than half reporting no use of alcohol in both the hurricanes (39%) and wildfires (41%) datasets, while no use of cannabis was reported by about 60% in both datasets (hurricanes dataset: 58%; wildfires dataset: 61%). The mean scores for alcohol and cannabis use among both datasets are also reported in Table 2. For the Hurricanes survey, mean scores for alcohol and cannabis use are reported for Florida, Georgia, North Carolina, and South Carolina, and potential differences in means were tested using analyses of variance (ANOVA). Results indicated no statistically significant differences among the four states in alcohol use, *F*(3, 1314) = 2.518, *p* > .05. However, the ANOVA was statistically significant for cannabis use, *F*(3, 1314) = 11.12, *p* < .001. North Carolina reported the highest mean cannabis use score (*M* = 3.07, *SD* = 2.22), followed by Florida (*M* = 2.43, *SD* = 2.14), South Carolina (*M* = 2.12, *SD* = 2.01), and Georgia (*M* = 2.12, *SD* = 2.01). Post hoc comparisons using the Bonferroni correction indicated that North Carolina differed significantly from Florida (*p* < .05), Georgia (*p* < .05), and South Carolina (*p* < .05), whereas Florida, Georgia, and South Carolina did not differ significantly from one another (*p* > .05).

**Table 2:** Descriptive statistics for alcohol and cannabis among participants.

	**Hurricanes survey (n = 1318)**	**Wildfires survey (n = 748)**
Alcohol use (Past 30 days)	0 days (39%)	0 days (40%)
	1–2 days (18%)	1–2 days (18%)
	3–5 days (16%)	3–5 days (16%)
	6–9 days (8.5%)	6–9 days (10%)
	10–19 days (9%)	10–19 days (7.5%)
	20–29 days (3.5%)	20–29 days (3.5%)
	Daily (6%)	Daily (5%)
Cannabis use (Past 30 days)	0 days (58%)	0 days (61%)
	1–2 days (9%)	1–2 days (7%)
	3–5 days (6.5%)	3–5 days (7%)
	6–9 days (6%)	6–9 days (7%)
	10–19 days (6%)	10–19 days (5.5%)
	20–29 days (2.5%)	20–29 days (3.5%)
	Daily (12%)	Daily (9%)
	**M(SD)**	**M(SD)**
Alcohol use (Past 30 days)		
Total sample	2.65 (1.83)	2.58 (1.76)
Florida	2.57 (1.84)	–
Georgia	2.53 (1.66)	–
North Carolina	2.91 (1.98)	–
South Carolina	2.66 (1.68)	–
	*F*(3, 1314) = 2.518, p > .05	
Cannabis use (Past 30 days)		
Total sample	2.47 (2.13)	2.34 (2.02)
Florida	2.43 (2.14)	–
Georgia	2.05 (1.83)	–
North Carolina	3.07 (2.22)	–
South Carolina	2.12 (2.01)	–
	*F*(3, 1314) = 11.12, p < .001	

Note: For the Hurricanes survey, mean scores for alcohol and cannabis use are reported for Florida, Georgia, North Carolina, and South Carolina, and potential differences in means were tested using analyses of variance (ANOVA).

### 3.2 Tests of hypotheses

We begin with the test for H1, which proposed that exposure to disaster-related information on social media would be associated with higher levels of depressive symptoms across our two datasets. For the hurricane dataset, the results shown on Table 3 did not reveal a significant relationship between exposure to disaster-related content via social media and levels of depression (b = 0.047, SE = 0.038, *p* = .216). By contrast, the results from the wildfires/Los Angeles dataset found that exposure to wildfire information from social media was positively associated with depressive symptoms (b = 0.118, SE = 0.04, *p* < .01). Hence, these results provide partial support for H1.

**Table 3:** Regression models for the association between exposure to disaster information on social media and depressive symptoms.

	Depressive symptoms (Hurricanes survey)	Depressive symptoms (Wildfires survey)
Age	−0.006(0.002)***	−0.007(0.002)**
Education	−0.010(0.015)	−0.029(0.019)
Income	−0.026(0.011)*	−0.024(0.012)*
Sex (Male)	−0.192(0.047)***	−0.067(0.062)
Race (White)	0.152(0.050)**	0.112(0.066)
Political ideology (Very liberal)	0.042(0.021)*	0.035(0.027)
Disaster-related stressors	0.199(0.014)***	0.130(0.021)***
Exposure to disaster information on other media	0.029(0.035)	0.127(0.044)**
Georgia vs. Florida	−0.115(0.071)	–
North Carolina vs. Florida	0.141(0.063)*	–
South Carolina vs. Florida	0.162(0.070)*	–
Exposure to disaster information on social media	0.047(0.038)	0.118(0.044)**
R^2^	0.251	0.250

Notes. Cell entries are unstandardized regression coefficients with standard errors in parentheses. For the hurricane data, we control for state of residence with Florida serving as the reference group. *** *p* < .001, ** *p* < .01, * *p* < .05.

Next, we examined H2, which assessed the association between exposure to disaster-related information on social media and the use of alcohol and cannabis in the two datasets. Our findings revealed robust support for our second hypothesis. When looking at the hurricane dataset, we found that exposure to hurricane content on social media was associated with higher levels of using both alcohol (b = 0.148, SE = 0.070, *p* = .04) and cannabis (b = 0.294, SE = 0.093, p < .01) (Table 4). These results were replicated in the wildfire dataset, with exposure to wildfire content on social media being associated with higher levels of using alcohol (b = 0.291, SE = 0.083, *p* < .001) and cannabis (b = 0.383, SE = 0.091, p < .001).

**Table 4:** Regression models for the associations between exposure to disaster information on social media and depressive symptoms with alcohol and cannabis use.

	Alcohol use (Hurricanes survey)	Cannabis use (Hurricanes survey)	Alcohol use (Wildfires survey)	Cannabis use (Wildfires survey)
Age	0.009(0.003)**	−0.009(0.003)**	0.003(0.004)	0.000(0.004)
Education	0.034(0.029)	−0.096(0.033)**	0.046(0.036)	−0.132(0.043)**
Income	0.132(0.023)***	0.065(0.026)*	0.065(0.024)**	0.009(0.024)
Sex (Male)	0.524(0.100)***	0.227(0.112)*	0.482(0.121)***	0.118(0.132)
Race (White)	−0.164(0.102)	−0.349(0.126)**	0.050(0.126)	0.176(0.147)
Political ideology (Very liberal)	−0.067(0.043)	0.017(0.049)	0.094(0.053)	0.058(0.054)
Disaster-related stressors	−0.020(0.030)	0.101(0.036)	0.028(0.039)	0.195(0.052)***
Exposure to disaster information on other media	0.206(0.070)**	−0.060(0.082)	0.202(0.088)*	0.090(0.096)
Georgia vs. Florida	−0.047(0.144)	−0.715(0.166)***	–	–
North Carolina vs. Florida	0.115(0.136)	0.294(0.161)	–	–
South Carolina vs. Florida	0.026(0.132)	−0.536(0.159)**	–	–
Exposure to disaster information on social media	0.148(0.070)*	0.294(0.093)**	0.291(0.083)***	0.383(0.097)***
Depressive symptoms	0.292(0.065)***	0.442(0.072)***	0.280(0.079)***	0.301(0.091)***
R^2^	0.141	0.164	0.224	0.215

Notes. Cell entries are unstandardized regression coefficients with standard errors in parentheses. For the hurricane data, we control for state of residence with Florida serving as the reference group. *** *p* < .001, ** *p* < .01, * *p* < .05.

H3 proposed that depressive symptoms would be associated with greater use of alcohol and cannabis. Similar to the results for H2, our findings showed support for our third hypothesis across both datasets. Higher levels of depressive symptoms were associated with higher levels of alcohol use (b = 0.292, SE = 0.065, p < .001) and cannabis use (b = 0.442, SE = 0.073, p < .001) in the hurricane dataset (Table 4). These results replicated in the wildfire dataset, with those reporting higher depressive symptoms being more likely to report using alcohol (b = 0.280, SE = 0.079, p < .001) and cannabis (b = 0.301, SE = 0.091, p < .001) (Table 4). Hence, these findings show robust support for H3.

Finally, H4 tested the indirect relationship of exposure to disaster information on social media and the use of the two substances (alcohol and cannabis) through depressive symptoms. Our findings for H4 revealed differential patterns across disaster types. For the hurricane dataset, no statistically significant indirect relationships were observed due to the lack of a significant association between exposure to hurricane content on social media and depressive symptoms, thereby precluding the mediation pathway. In contrast, results from the wildfire dataset revealed that exposure to wildfire information on social media was associated with reporting higher depressive symptoms, and depressive symptoms were then associated with higher use of alcohol (b = 0.033, 95% CI 0.010, 0.075) and cannabis (b = 0.035, 95% CI 0.010, 0.084). These results provide some support for H4.

## 4 Discussion

The present study sought to examine how exposure to disaster-related content on social media is associated with both depressive symptoms and post-disaster use of alcohol and cannabis. This study also aimed to analyze a potential mediation model whereby exposure to disaster-related content on social media is positively associated with the use of the two substances through higher levels of depressive symptoms. We analyzed survey data collected during the aftermath of two distinct, though major, types of natural disasters: Hurricanes Helene and Milton in 2024 and the LA County wildfires in 2025. Our study's contribution to the current literature can be summarized along three key areas.

First, our study provided evidence that being exposed to disaster-related information on social media is associated with reporting higher levels of depressive symptoms. However, this association was significant only among those who had experienced the LA County wildfires, but not among those who experienced Hurricane Helene and/or Hurricane Milton. On the surface, these findings imply that wildfire content on social media may be more impactful than hurricane content as a factor associated with heightened levels of depressive symptoms. We interpret these divergent results between the wildfire and hurricane datasets in light of the findings for our mediation model, which we discuss below. Nevertheless, these findings help extend previous work on the link between exposure to disaster information more broadly and psychological distress (Garfin et al., 2022; Houston et al., 2018; Thompson et al., 2019).

Second, we found that being exposed to natural disasters on social media and depressive symptoms were both positively associated with post-disaster use of alcohol and cannabis. These relationships were observed in both the hurricane and wildfire datasets. These findings reinforce past studies showing that social media use is positively associated with alcohol consumption (Alhabash et al., 2022) and cannabis initiation (e.g., Kelleghan et al., 2021). Our results help contribute to this line of work by providing evidence that indirectly experiencing hurricanes and wildfires through social media could be associated with greater post-disaster substance use. Furthermore, our results confirm extant research that has established a linkage between psychological distress and post-disaster use of substances (e.g., Flory et al., 2009; Noel et al., 2021).

Yet we point out that the literature reviewed above suggests that hurricane-related social media content tends to center on themes such as price gouging, looting, and environmental vulnerability (Sovacool et al., 2020), as well as property damage and infrastructure loss (Shin et al., 2012). While such content can be distressing, it may be less immediately psychologically activating than the graphic, unfiltered imagery and personal loss narratives more commonly associated with wildfire coverage. Evidence from wildfire-related social media research indicates that damage-focused content is particularly likely to be amplified and shared (Stimpson et al., 2025), suggesting that the most emotionally intense material tends to circulate more widely in the context of wildfires than hurricanes. Hurricane-related posts, by contrast, may more often focus on preparatory activities, flooding aftermath, or infrastructure disruption — content that, while concerning, may not evoke the same level of immediate psychological distress as the visceral destruction imagery characteristic of wildfire social media coverage. However, we note that our survey did not include measures that would have allowed us to assess the specific and nuanced types of content in disaster-related messages (i.e., hurricanes, wildfires) that participants were exposed to on social media platforms. Because of this limitation, we could only the above to be the case for social media content portraying the hurricanes and wildfire. Therefore, more research is needed to thoroughly examine potential differences of exposure to distinct types of natural disasters.

Third, our findings provide some support for our proposed mediation model. That is, we found evidence that exposure to wildfire content on social media is positively linked with using alcohol and cannabis through the pathway of depression. However, this same mediation process did not occur in the hurricanes dataset, indicating that exposure to hurricane content on social media was not associated with alcohol and cannabis use through depression. This pattern of results can be aligned with our set of findings that tested the association between exposure to disaster information on social media and depressive symptoms. These differential patterns suggest that the psychological impact of disaster-related social media content may depend on characteristics specific to the disaster type or the nature of content typically shared during different disasters.

Thus, our findings imply that not all disaster-related social media content may produce equivalent psychological responses. This challenges assumptions about uniform media effects and highlights the need for more nuanced theoretical models that account for disaster-specific content characteristics, temporal factors, or audience vulnerabilities. One explanation is that wildfire-related social media content may include more visually dramatic and emotionally intense imagery, such as flames consuming homes or apocalyptic orange skies, that could generate stronger vicarious trauma responses than hurricane content. Conversely, hurricane-related posts may focus more on preparation activities, flooding aftermath, or infrastructure damage, which, while concerning, may not evoke the same immediate psychological distress. Therefore, more research is needed to thoroughly examine potential differences of exposure to distinct types of natural disasters.

Finally, our mediation-based findings help to augment Alexander and Ward's (2018) conceptual model for post-disaster substance use. As explained above, Alexander and Ward's model conceptualized psychological distress, such as depression, as a central pathway through which direct exposure to disasters leads to increased substance use.

## 5 Strength and limitations

The larger contribution of the present study is that it extends this line of work by providing evidence that indirect exposure to disasters through social media could serve a similar role to direct experiences with natural disasters. Yet while our study contributes to the literature, we should acknowledge weaknesses with our data that may have affected our results. First, it is important to note that one weakness with our study is our reliance on cross-sectional data. As with any research using cross-sectional data, follow-up research, particularly experimental studies and multi-wave survey data, should be done to assess if the proposed paths work when assessing causality. Second, because our data were not collected using probability-based sampling, they are not representative of the populations under study. We thus recommend that future studies utilize representative samples. Third, given that our data were collected in the areas impacted by the disasters tied to our surveys, the participants likely still experienced the disasters directly. However, we should emphasize that we included disaster-related stressors, an indicator of direct exposure, as a covariate in our models. Fourth, as mentioned, our survey did not include measures that would have allowed us to assess the types of content in disaster-related messages (i.e., hurricanes, wildfires) that participants encountered on social media platforms.

## 6 Conclusion

The results of our study highlight the larger impacts that natural disasters could have on people living in the path of these events. Namely, for those who directly experience a disaster, there is the potential that using social media for news or information about the disaster may serve as a risk factor associated with heightened distress and substance use. Such findings have implications for the prevention of substance use in the aftermath of disasters. For instance, those who directly experience a disaster could be given priority in terms of resources to help them deal with their losses, which may help to buffer or mitigate post-disaster distress and the potential for substance use. However, it may be important for institutions to offer some help to the broader public, when appropriate, to help others deal with the stress of living through a natural disaster. Furthermore, as recent research has shown that social media users may be more likely to engage in content highlighting the impacts and damage tied to a disaster (Stimpson et al., 2025), given the results of our study, we highlight the importance of greater thoughtfulness about what is being covered and how emphasizing the specific aspects of natural disasters could be influencing others.

## CRediT authorship contribution statement

**Francis Dalisay:** Writing – review & editing, Writing – original draft, Resources, Project administration, Methodology, Investigation, Formal analysis, Data curation, Conceptualization. **Jay Hmielowski:** Writing – review & editing, Writing – original draft, Formal analysis, Conceptualization. **Young-Rock Hong:** Writing – review & editing, Writing – original draft.

## Declaration of competing interest

The authors declare that they have no known competing financial interests or personal relationships that could have appeared to influence the work reported in this paper.

## Data Availability

Data will be made available on request.

## Appendix A Supplementary data

Supplementary material 1 CONSORT diagram for participant flow through data collection for hurricanes survey.

Supplementary material 2 CONSORT diagram for participant flow through data collection for wildfires survey.
