# Supplementary material for: Exposure to disaster information on social media, depressive symptoms, and alcohol and cannabis use in the aftermath of two natural disasters
Source: Addict Behav Rep. 2026 Jun 24;24:100723. doi: 10.1016/j.abrep.2026.100723 (PMC13321261; doi:10.1016/j.abrep.2026.100723)
Supplement: Supplementary material 1 — CONSORT diagram for participant flow through data collection for hurricanes survey. [file mmc1.pdf]

Hurricanes Survey — Participant Flow

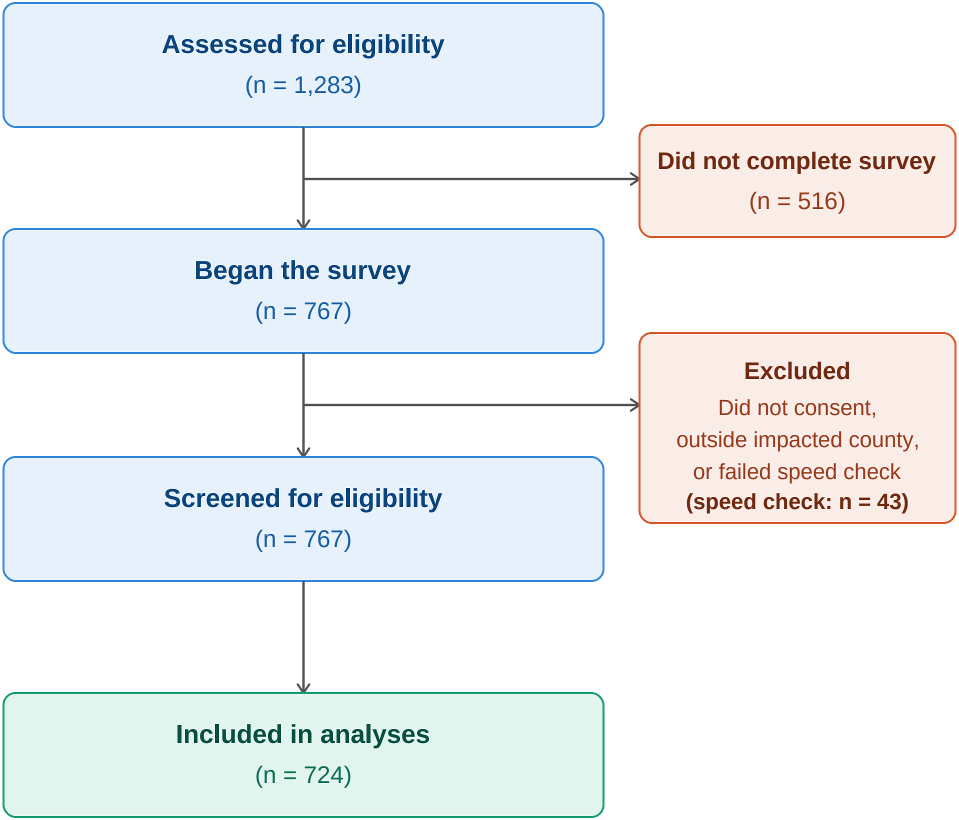

Speed check threshold: completion time less than 9 minutes (median survey duration)
